# Supplementary material for: Early-stage bifurcation of crystallization in a sphere
Source: Nat Commun. 2023 Aug 31;14:5299. doi: 10.1038/s41467-023-41001-6 (PMC10471623; doi:10.1038/s41467-023-41001-6)
Supplement: Supplementary file 1 — Supplementary Information [file 41467_2023_41001_MOESM1_ESM.pdf]

Supplementary Information for

Early-Stage Bifurcation of Crystallization in a Sphere

Chrameh Fru Mbah[1,†], Junwei Wang[2,†], Silvan Englisch[3,†], Praveen Bommineni[1,4], Nydia Roxana Varela-Rosales[1], Erdmann Spiecker[3], Nicolas Vogel[2*], and Michael Engel[1*]

[1]Institute for Multiscale Simulation, IZNF, [2]Institute of Particle Technology, [3]Institute of Micro- and Nanostructure Research and Center for Nanoanalysis and Electron Microscopy, IZNF, Friedrich-Alexander-Universität Erlangen-Nürnberg, 91058 Erlangen, Germany, [4]Department of Chemical Engineering, National Institute of Technology Warangal, Telangana-506004, India

[†] These authors contributed equally.

[*] Corresponding authors: nicolas.vogel@fau.de, michael.engel@fau.de

Supplementary Table

	Q_4	Q_6	Q_8	Q_{12}
**Anti-Mackay**	0.00	0.07	0.00	0.42
**Mackay**	0.00	0.21	0.00	0.50
**Decahedron**	0.04	0.33	0.17	0.52
**FCC**	0.19	0.57	0.40	0.60

Supplementary Table S1. Rotationally invariant global bond orientational order parameters calculated for the four ideal cluster models in Supplementary Figure S6.

Supplementary Figures

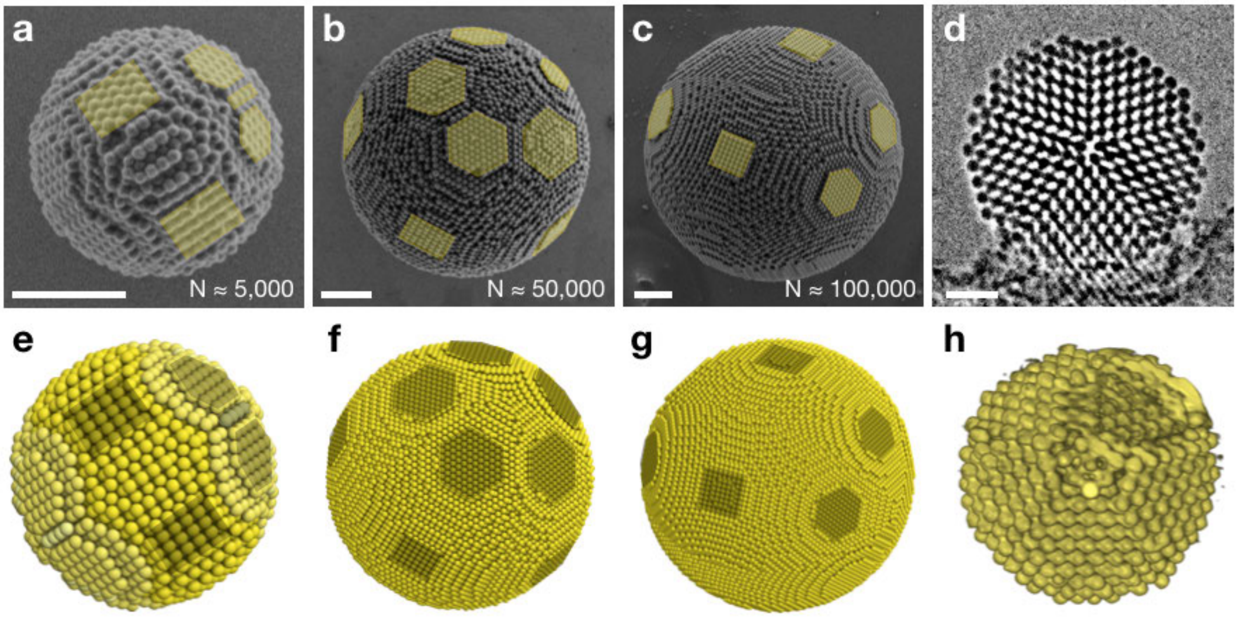

Supplementary Figure S1. a-c, Scanning electron microscope images of decahedral colloidal clusters crystallized by slow drying of polystyrene particle in aqueous emulsion droplets. Cluster sizes range from 5,000 to 100,000 particles. The surface is tiled characteristically by ten (111) and five (100) crystal planes highlighted in the images by yellow hexagons and squares, respectively. Only one five-fold symmetric axis is visible in (b) due to the specific viewing angle. **e-g**, Corresponding geometric models of the decahedral clusters in (a-c). **d**, X-ray image revealing multi-twinned crystalline grains sharing one five-fold axis in the decahedral cluster. **h**, X-ray tomography acquired individual particle positions demonstrating the presence of five (111) planes at the surface. Colloidal particle diameter, 230 nm in (a-c), 500 nm in (d). Scale bars, 2 µm.

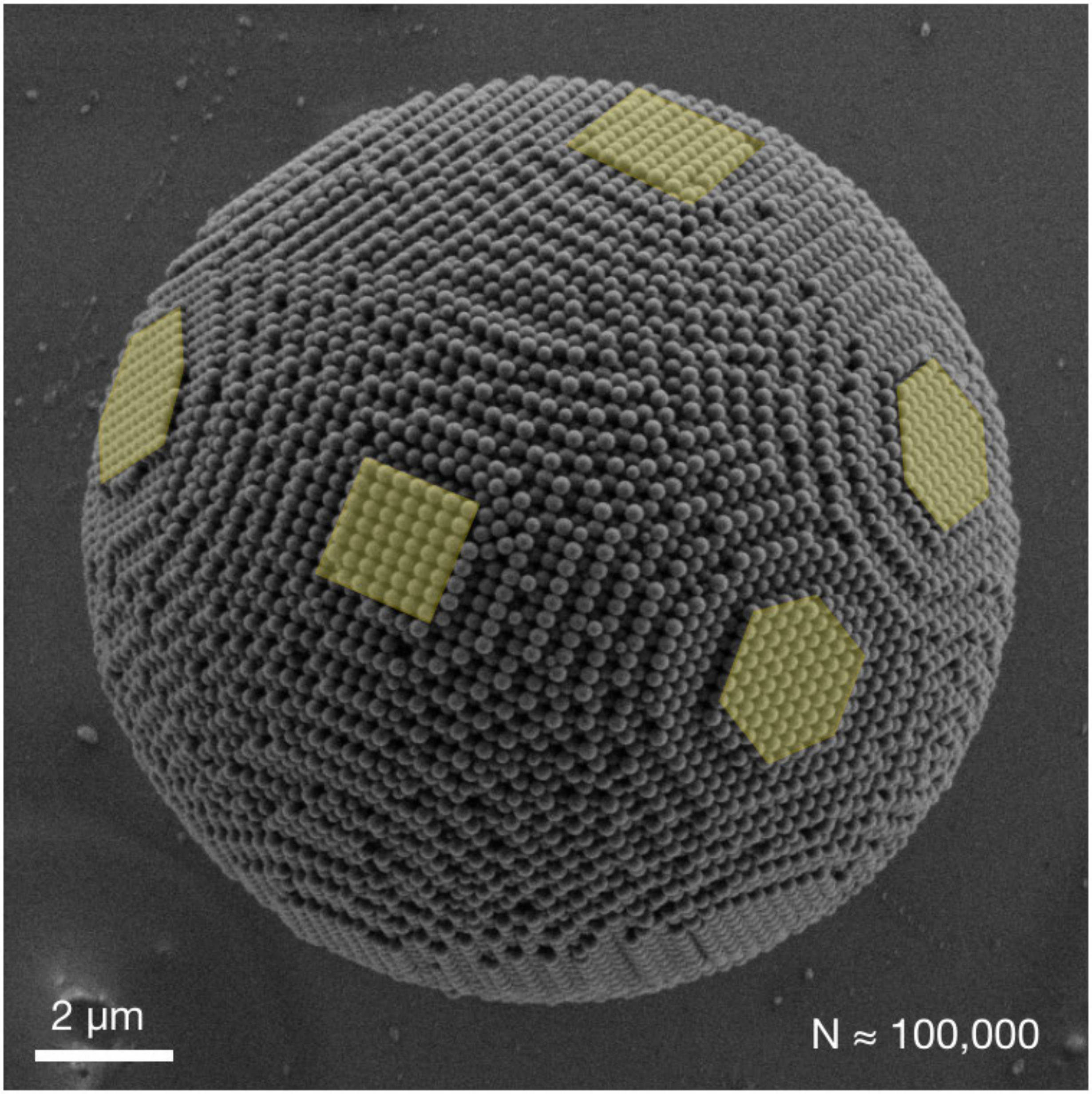

Supplementary Figure S2. Scanning electron microscope (SEM) image of a large decahedral colloidal cluster. (111) and (100) crystal planes at the surface are highlighted by yellow hexagons and squares, respectively. A grain boundary separating two neighboring grains as well as the presence of large surface areas of particles with low coordination number are clearly visible. This cluster is also show in Supplementary Figure S1c.

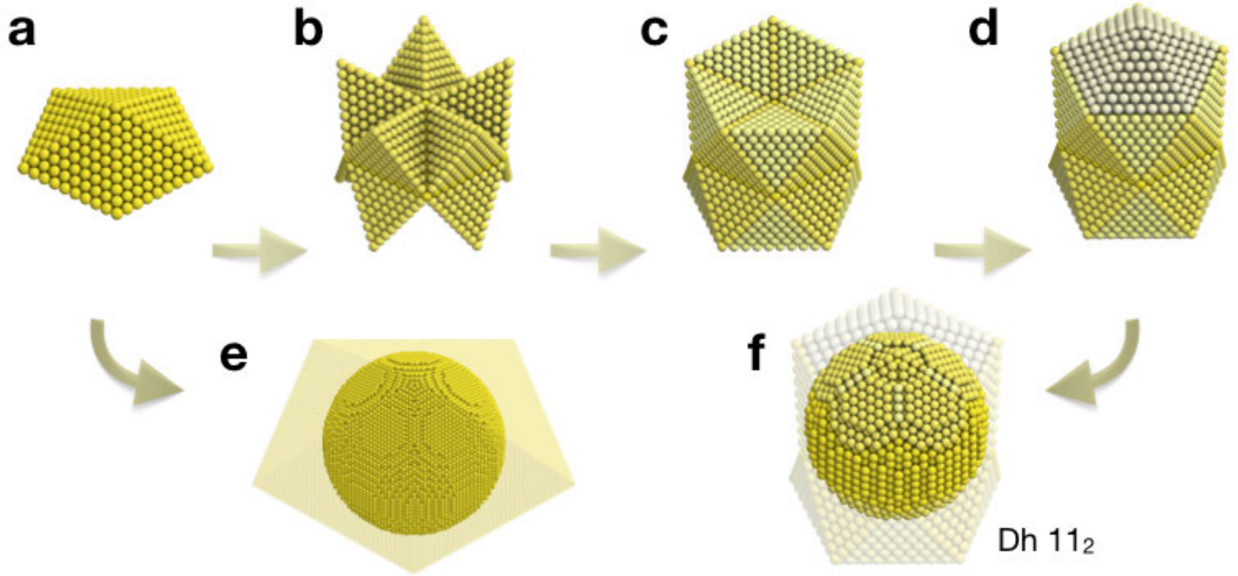

Supplementary Figure S3. Structure model for decahedral colloidal clusters. **a**, Pentagonal bipyramid model made from equal-sized spheres. Five grain in slightly deformed face-center cubic (fcc) arrangement twinned with its neighbors around a common five-fold axis. The arrangement is equivalent to sequential addition of shells over a seven-sphere pentagonal bipyramid. This is the basic of Marks' and Ino's decahedron model[1,2]. **b**, Ten slightly deformed tetrahedral fcc grains can be added over the faces of the pentagonal bipyramid. **c**, Ten additional fcc grains can be added over the edges of the pentagonal bipyramid. **d**, Ten Additional grains can be added over the vertices of the pentagonal bipyramid to complete the extended decahedron model. **e**, Spherical truncation of the extended decahedron model removes the particles outside a certain radius mimicking the effect of droplet confinement and produces decahedral clusters with quasi-spherical shape. Apart from (111) and (100) planes, the surface has a large area with low coordination number that does not exhibit a close-packed surface tiling near the vertices of the pentagonal bipyramid. **f**, Spherical truncation of the extended model (shown in d) produces decahedral clusters with close-packed surface features and defined twinning, similar to anti-Mackay surface features in icosahedral clusters. The cluster structure can be denoted as 11_2, i.e., eleven shells in total and two surface layers twinned with the interior[3]. This structure coincides with the experimental observations in Figure 1a and Supplementary Figure S1a. Decahedral clusters with anti-Mackay type surface restructuring (f) are observed at relatively small cluster size. We expect the transition to be similar to that observed in icosahedral clusters, which occurs around radius 15σ.

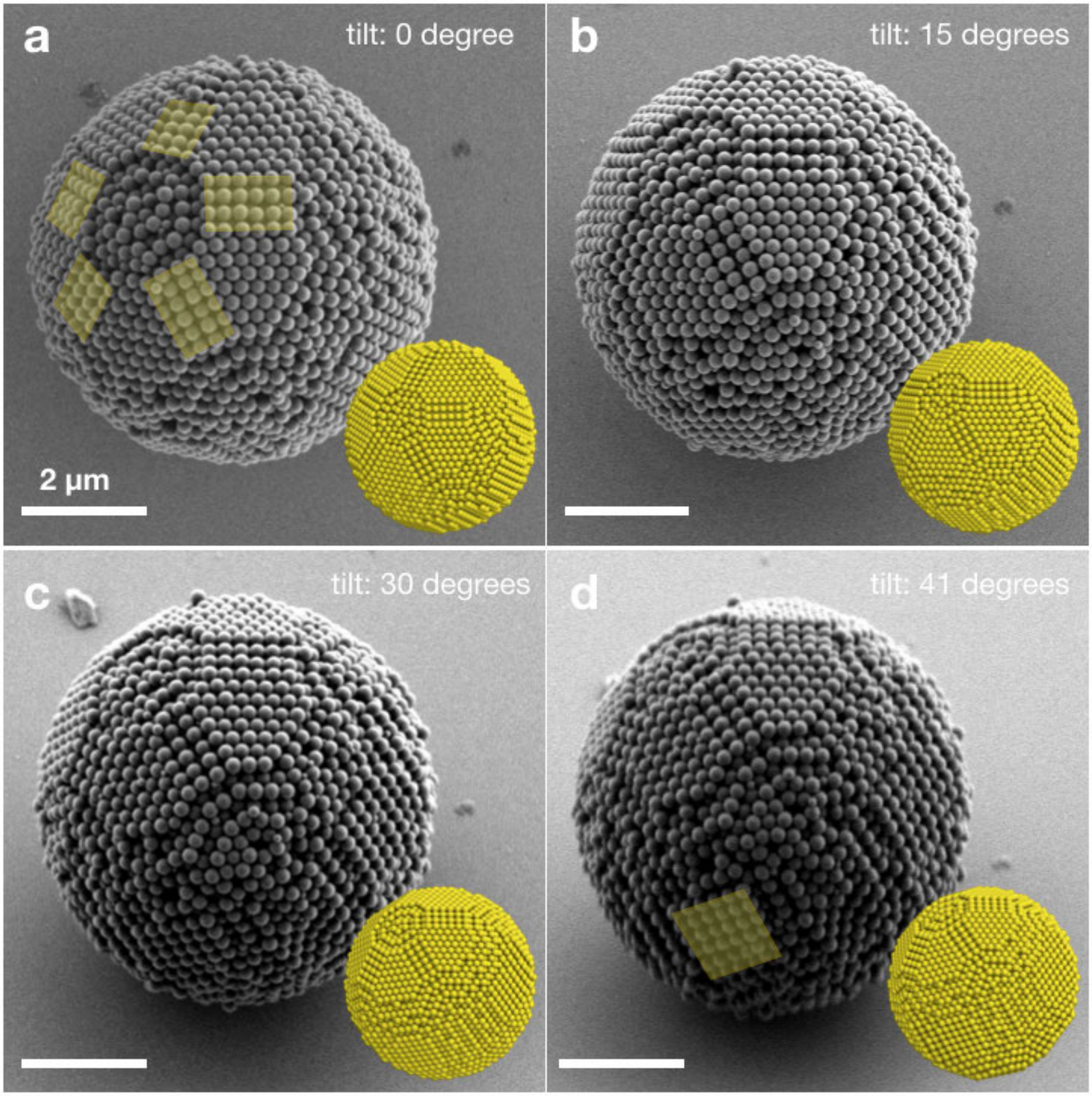

Supplementary Figure S4. SEM images of a decahedral colloidal clusters with anti-Mackay layer-like surface structures, similar to icosahedral clusters. Only one five-fold symmetri axis is found in all tilted images. The lack of other five-fold axes allows us to distinguish the decahedral cluster from an icosahedral cluster. The sample stage is tilted up to 41 degrees in the SEM to show the side view of the cluster. The characteristic rectangular (100) regions due to surface twinning is marked in yellow in **a**. The width of the rectangular region consists of four particles, indicating 3 twinning layers at the surface, in agreement with model (inset). **b-d**, The substrate is tilted to reveal the side view of the cluster with square (100) regions.

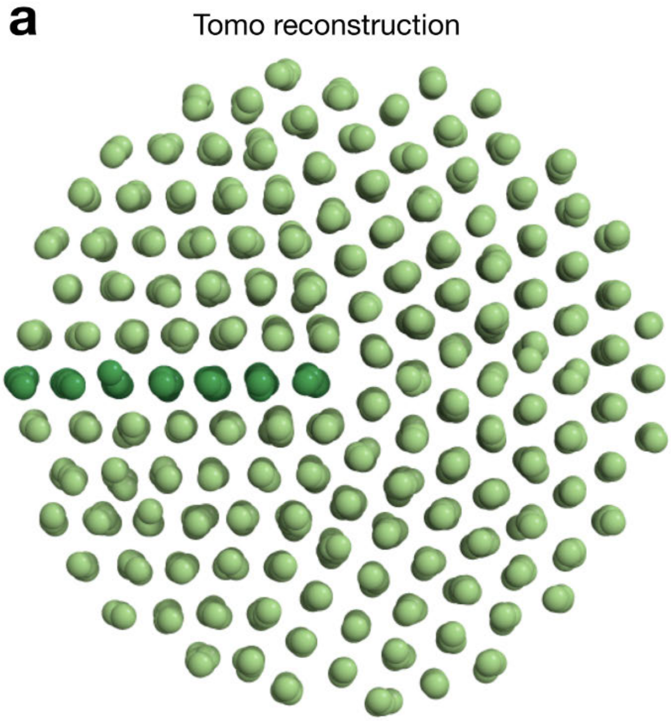

Supplementary Figure S5. Particles positions of a decahedral colloidal cluster viewed along the five-fold axis extracted from X-ray tomography (tomo) reconstruction (2286 particles). Particles are shrunk in size for visual clarity. A planar defect extending radially from the five-fold axis is marked in dark green.

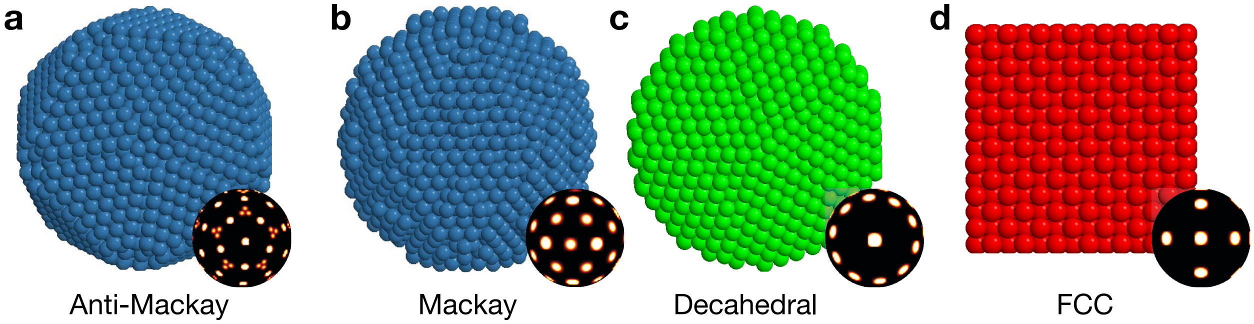

Supplementary Figure S6. Ideal cluster models of **a**, anti-Mackay icosahedral cluster, **b**, Mackay icosahedral cluster, **c**, decahedral cluster, and **d**, FCC cluster (shown is a cubic patch of the cluster). The bond-orientational order diagram shows the typical signatures of the associated clusters that we use for quickly classifying the cluster type from a simulation outcome.

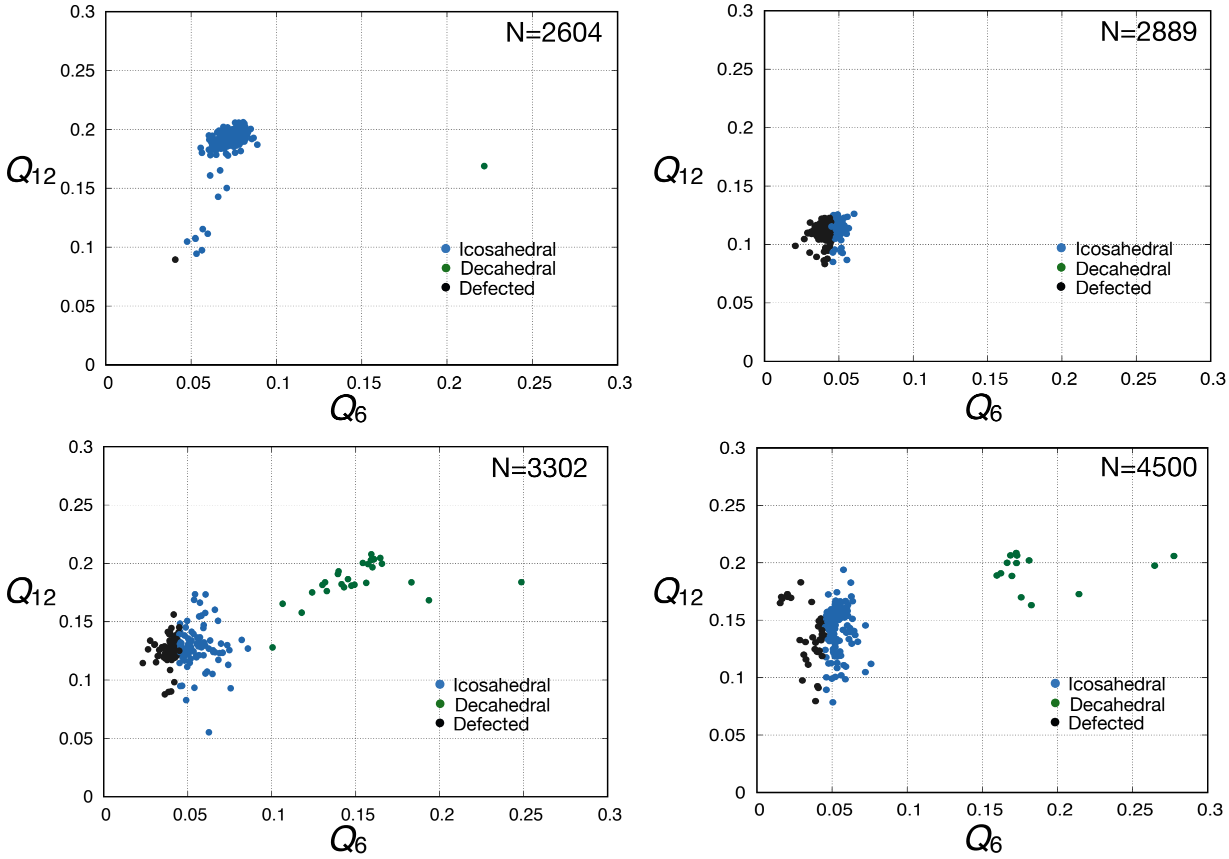

Supplementary Figure S7. Distributions of the global bond orientational order parameters Q_6 and Q_{12} calculated for clusters obtained in 192 simulations each at four system sizes. Q_6 is best suited to distinguish icosahedral and decahedral clusters. Candidate decahedral clusters have $Q_6 > 0.1$, candidate icosahedral clusters $0.04 < Q_6 < 0.1$. In the range $Q_6 < 0.04$, clusters might still exhibit weak icosahedral symmetry but are typically highly defected. We do not classify such clusters as icosahedral clusters in Figure 2. Simulations at $N = 2604$ ($R = 8.6\sigma$ in Figure 2) form icosahedral clusters reliably. Simulations at $N = 2889$ ($R = 8.8\sigma$) form predominantly defected clusters. Simulations at $N = 3302$ ($R = 9.2\sigma$) form a comparably large number of decahedral clusters. Simulations at $N = 4500$ ($R = 10.2\sigma$) form a mix of icosahedral and decahedral clusters.

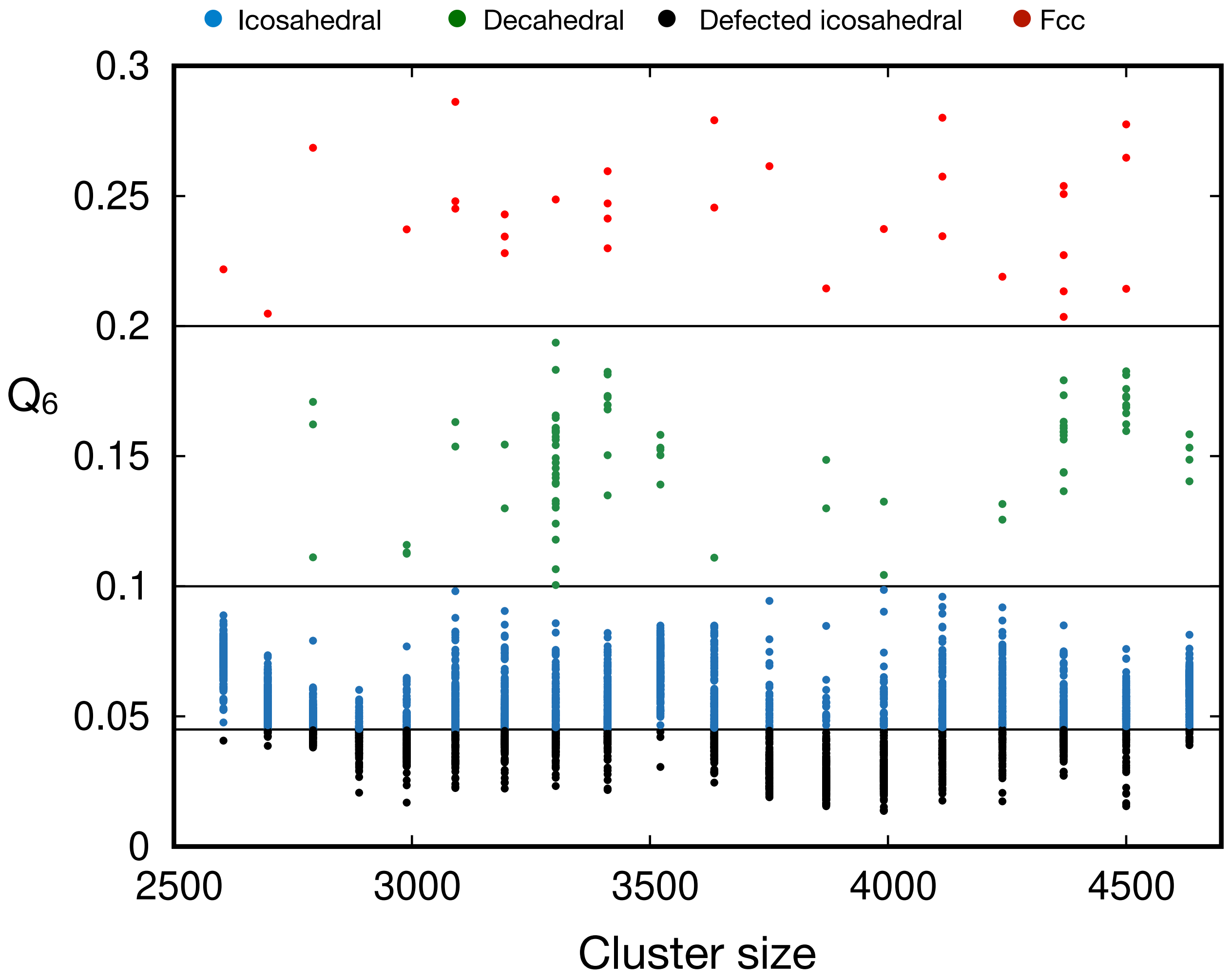

Supplementary Figure S8. Global bond orientational order parameters Q_6 for all crystallization simulations. This data forms the basis for the statistical analysis of icosahedral and decahedral cluster occurrence in Figure 2b,c. The candidate icosahedral and decahedral clusters classified in this way are further analyzed manually and with the help of point group symmetry quantification.

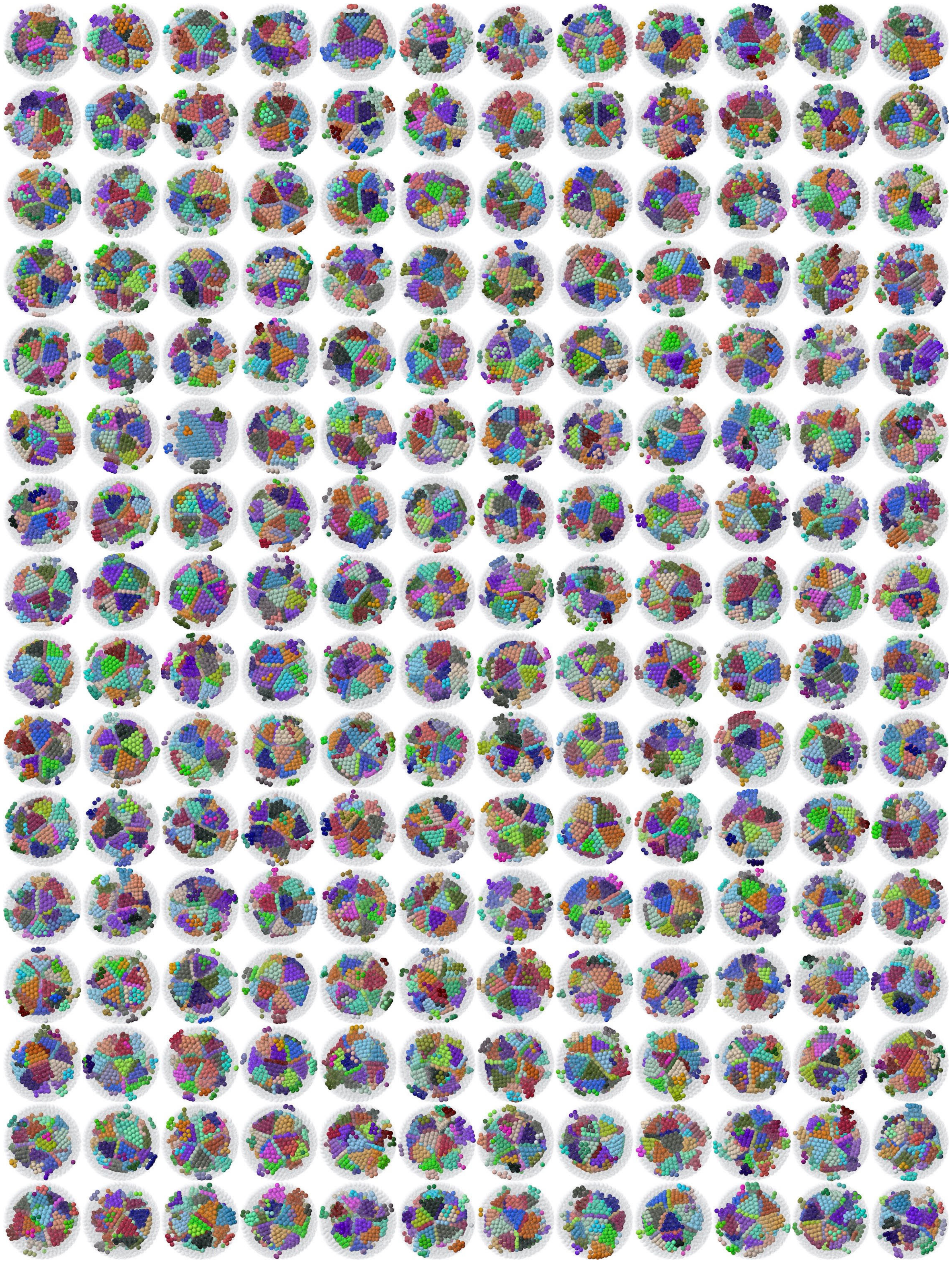

Supplementary Figure S9. Clusters with grains extracted from 192 simulations at system size $N = 2604$ ($R = 8.6\sigma$ in Figure 2). Spheres not belonging to crystalline grains are shown semi-transparent gray. The majority of clusters are icosahedral clusters (Supplementary Figure S10).

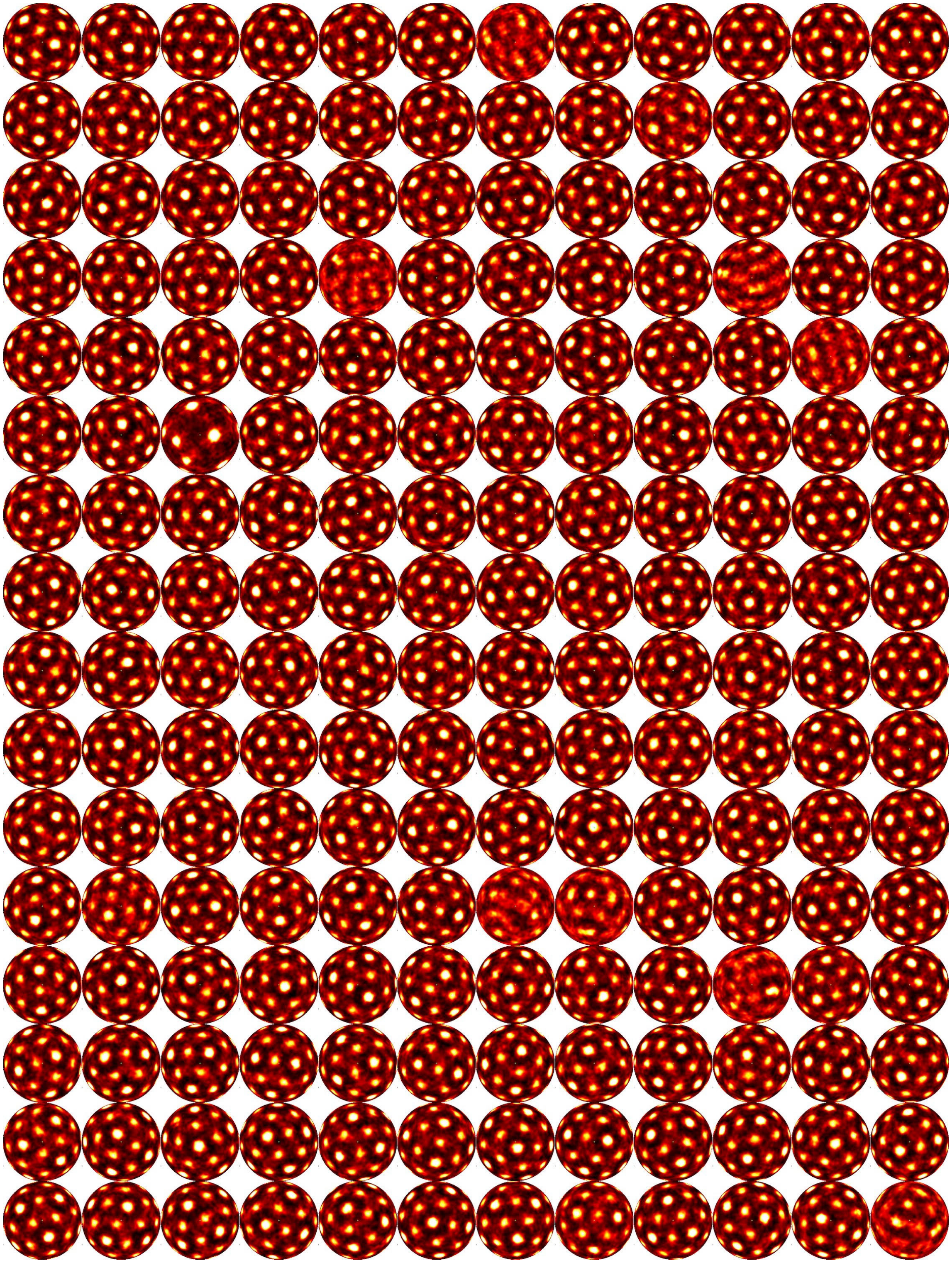

Supplementary Figure S10. Bond-orientational order diagrams for system size $N = 2604$. This number of particles corresponds to a minimum in the icosahedral free energy-curve. A strong tendency to icosahedral symmetry is apparent with only nine non-icosahedral clusters (including one rare fcc/hcp cluster).

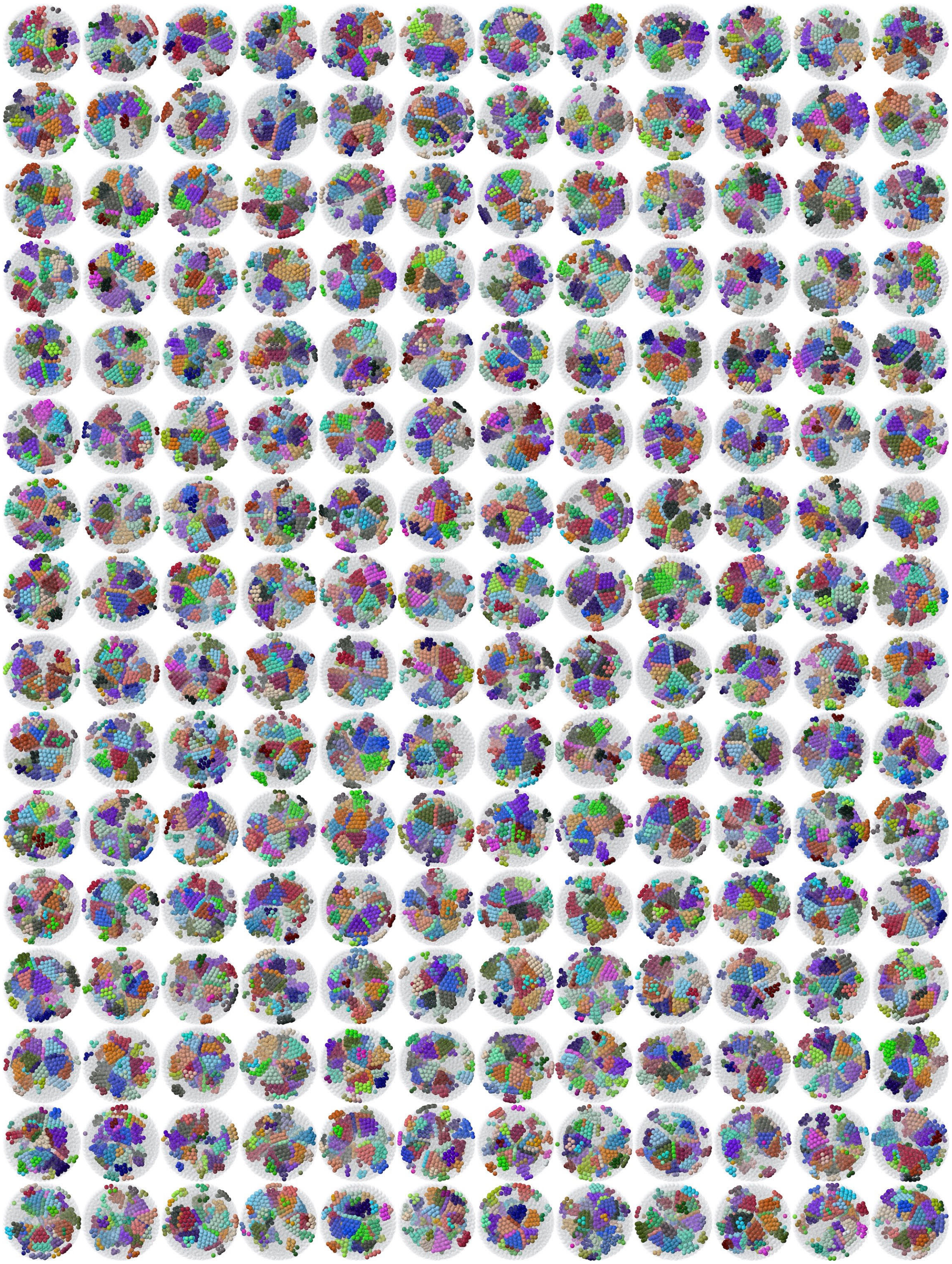

Supplementary Figure S11. Clusters with grains extracted from 192 simulations at system size $N = 2889$ ($R = 8.8\sigma$ in Figure 2). Spheres not belonging to crystalline grains are shown semi-transparent gray. The majority of clusters are defected (Supplementary Figure S12).

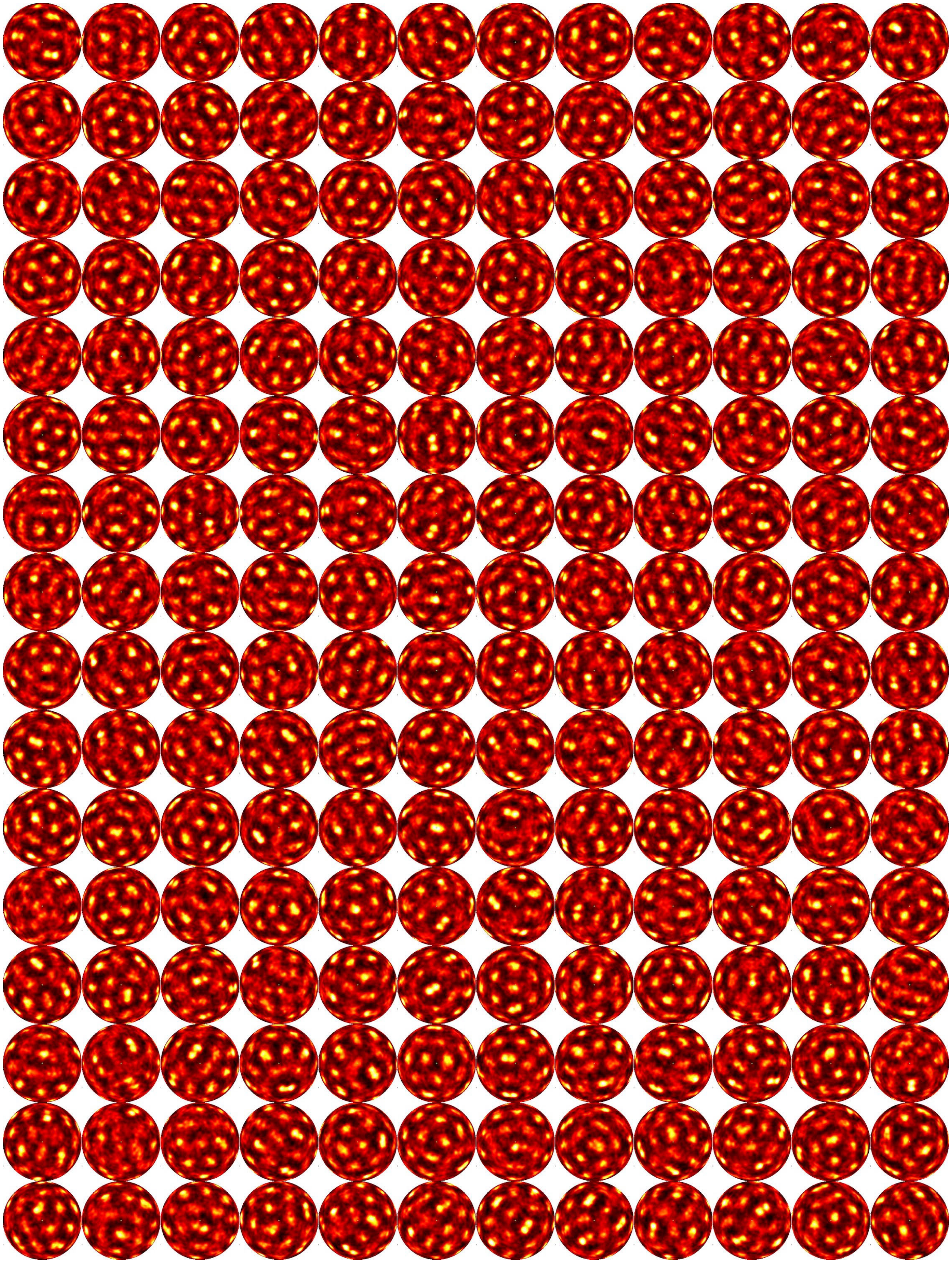

Supplementary Figure S12. Bond-orientational order diagrams for system size *N* = 2889. At this number of particles in an off-magic number region, defected clusters with partially-broken icosahedral symmetry are prevalent.

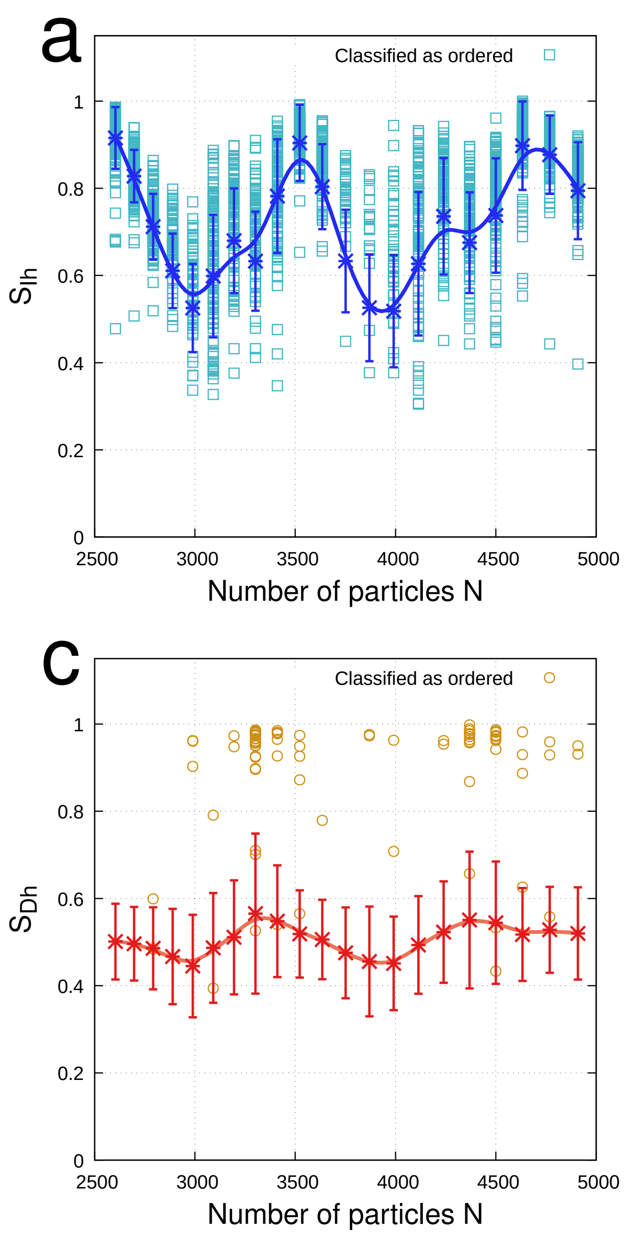

Supplementary Figure S13. Point group symmetry quantifiers **a,b**, for icosahedral order, S_{Ih} and **c,d**, for decahedral order, S_{Dh}. Data points (symbols) are for 192 independent simulations each at the system sizes studied in Figure 2. We distinguish clusters classified by manual inspection as ordered with icosahedral order (a), as ordered with decahedral order (c), and not classified as ordered (b,d). The line shows the average over all simuation outcomes with standard deviation error bars. S_{Dh} identifies decahedral clusters well. S_{Ih} has problems distinguishing icosahedral clusters and defected clusters with defect wedges that break icosahedral symmetry. This is why we must still rely on some manual classification for the distinction of icosahedral clusters and defected icosahedral clusters but not for the classification of decahedral clusters.

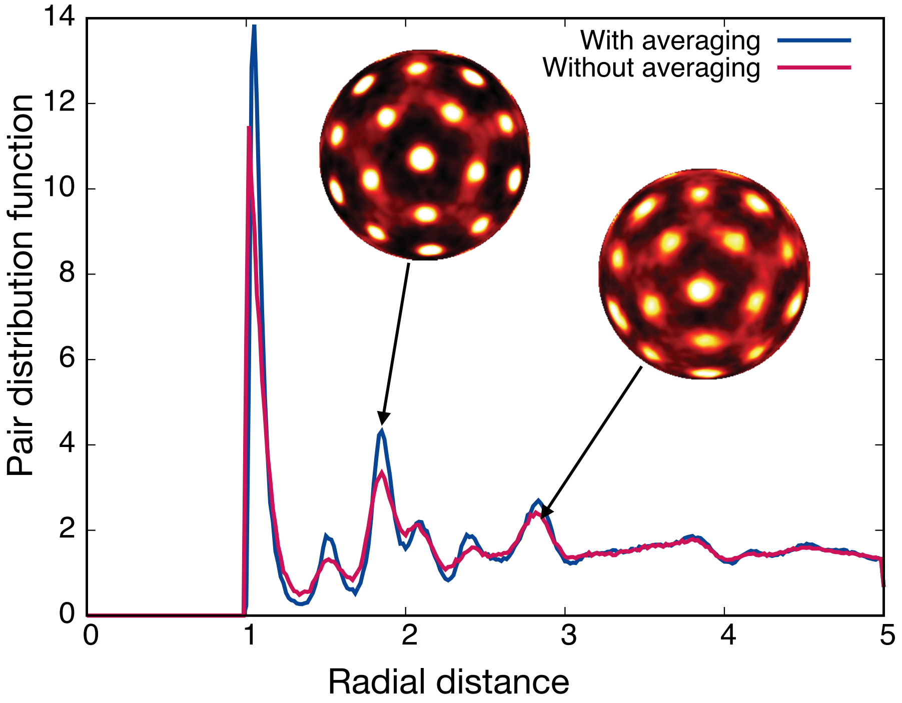

Supplementary Figure S14. The pair distribution function (also known as radial distribution function) of an icosahedral cluster crystallized in a simulation of 5781 hard spheres. The pair distribution function is weakly affected by short-time averaging of particle positions that we apply to reduce thermal noise. Peak height increases and peak width decreases by averaging because the vibrations are suppressed. Noise reduction leads to a significant improvement in the characterization of structural order (higher uniformity). This is apparent in the bond-orientational order diagrams (insets). Radial distance is measured in units of σ.

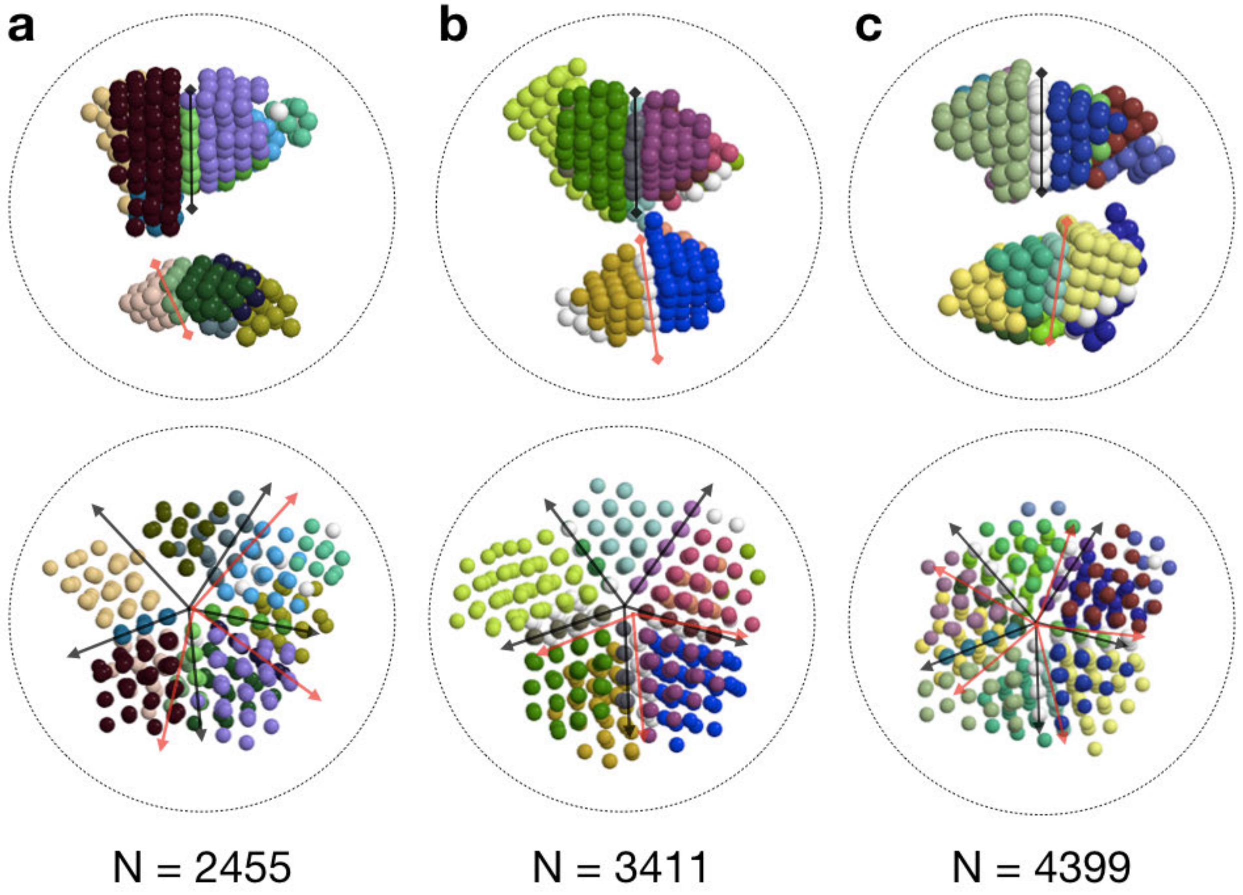

Supplementary Figure S15. Grain alignment during simultaneous nucleation of two pentagonal bipyramid nuclei at antipodes near the interface. Grains are colored randomly. Fluid particles and unstable grains are not shown. **Top row** (side view), Simultaneous nucleation occurs occasionally. Two pentagonal bipyramid nuclei form. Their symmetry axes are aligned with an angle smaller than the 36° staggering angle between upper and lower five-fold patterns in an icosahedron. The twinning axes in the upper and lower nuclei are marked by black and red arrows. **Bottom row** (five-fold view), Black arrows mark the projection of the twin planes the upper five grains, red arrows the projection of twin planes in the lower grains. The nuclei align their symmetry axes without being in contact across the fluid. As crystallization proceeds, one of the nuclei either melts, or they align completely to the antipodes. In the latter case, the two nuclei eventully merge at the center as they grow.

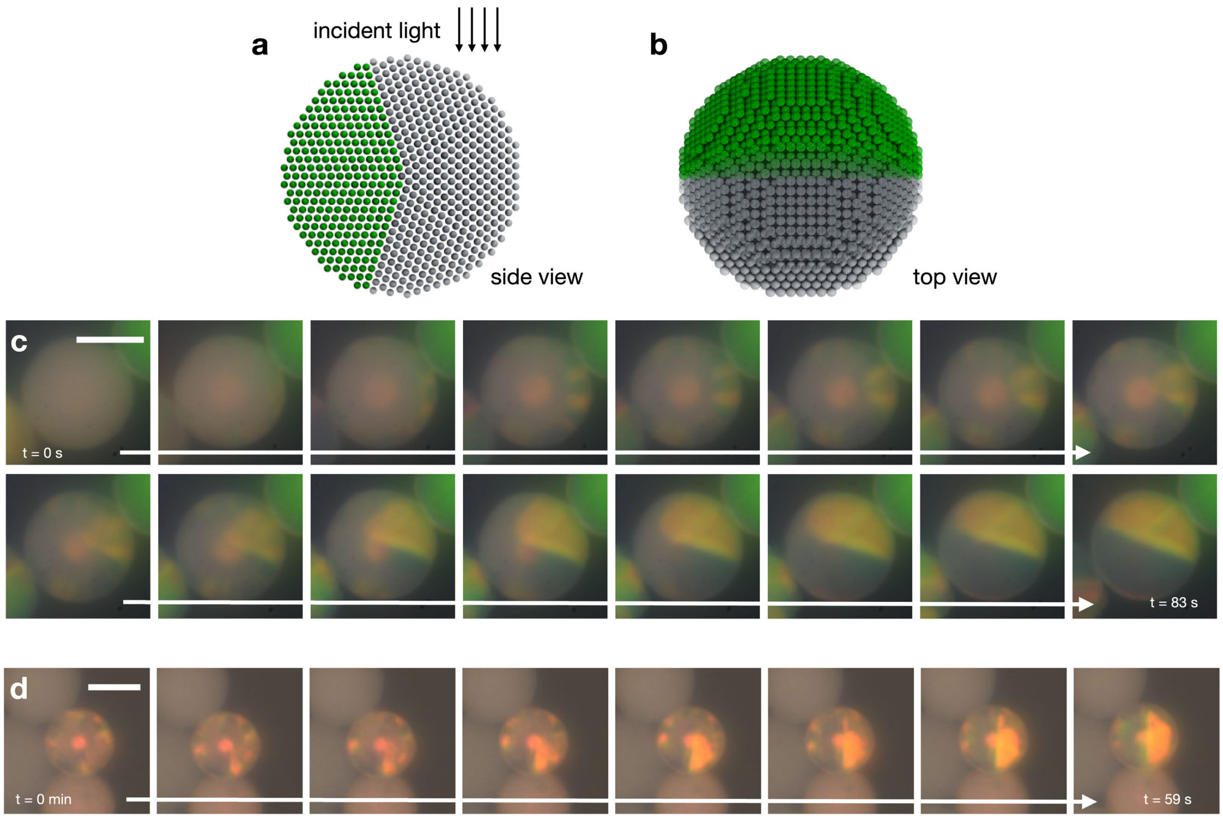

Supplementary Figure S16. Decahedral colloidal cluster formation in drying droplets. **a,b**, Decahedral cluster model. The grains marked in green align their (111) planes perpendicularly to the incident light in the microscope giving rise to a semicircle structural color motif[4]. **c**, Observation in real time under an optical microscope in reflection mode with an 125x objective (Supplementary Movie S5). The whitish appearance in the droplet results from random scattering of 230 nm PS particles in the liquid state. As time advances (indicated by white arrows), crystalline regions can be seen at the rim of the droplet. A central circle appears with dim red hue suggesting formation of onion ring-like pre-ordered fluid near the droplet interface. A growing triangular region in yellow extend from the rim to the center, then pass the center towards the other side, eventually completing the semicircle. The yellow color result from the Bragg diffraction of the colloidal crystal formed inside the droplet. Meanwhile, the central circle disappears, the lower semicircle becomes transparent suggesting the disappearance of the onion ring-like layers and the crystallization in the entire volume of the droplet. **d**, Another observation with a 40x microscope objective (Supplementary Movie S6) suggest the same grain development in decahedral cluster formation. It is suspected that the color difference between (c) and (d) results from different packing fraction of particles in the droplet at the onset of crystallization as well as the change in numerical aperture of the objective. When droplet drying completes, both clusters appear green. This color is determined by the diameter of the colloidal particles. All scalebars, 10 µm.

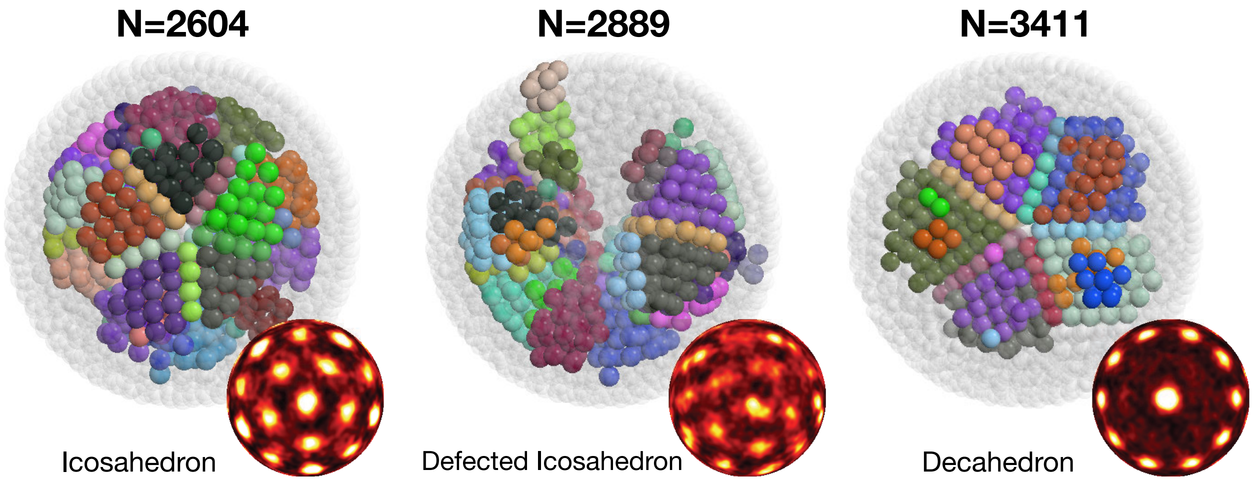

Supplementary Figure S17. Clusters obtained from event-driven molecular dynamics simulation. The cluster $N = 2604$ is an icosahedral cluster belonging to a magic number region. The cluster $N = 2889$ belongs to an off-magic number region and forms a defective cluster with partially broken icosahedral symmetry. Defects accumulate in a gap between the tetrahedral grains. The cluster $N = 3411$ is a decahedral cluster.

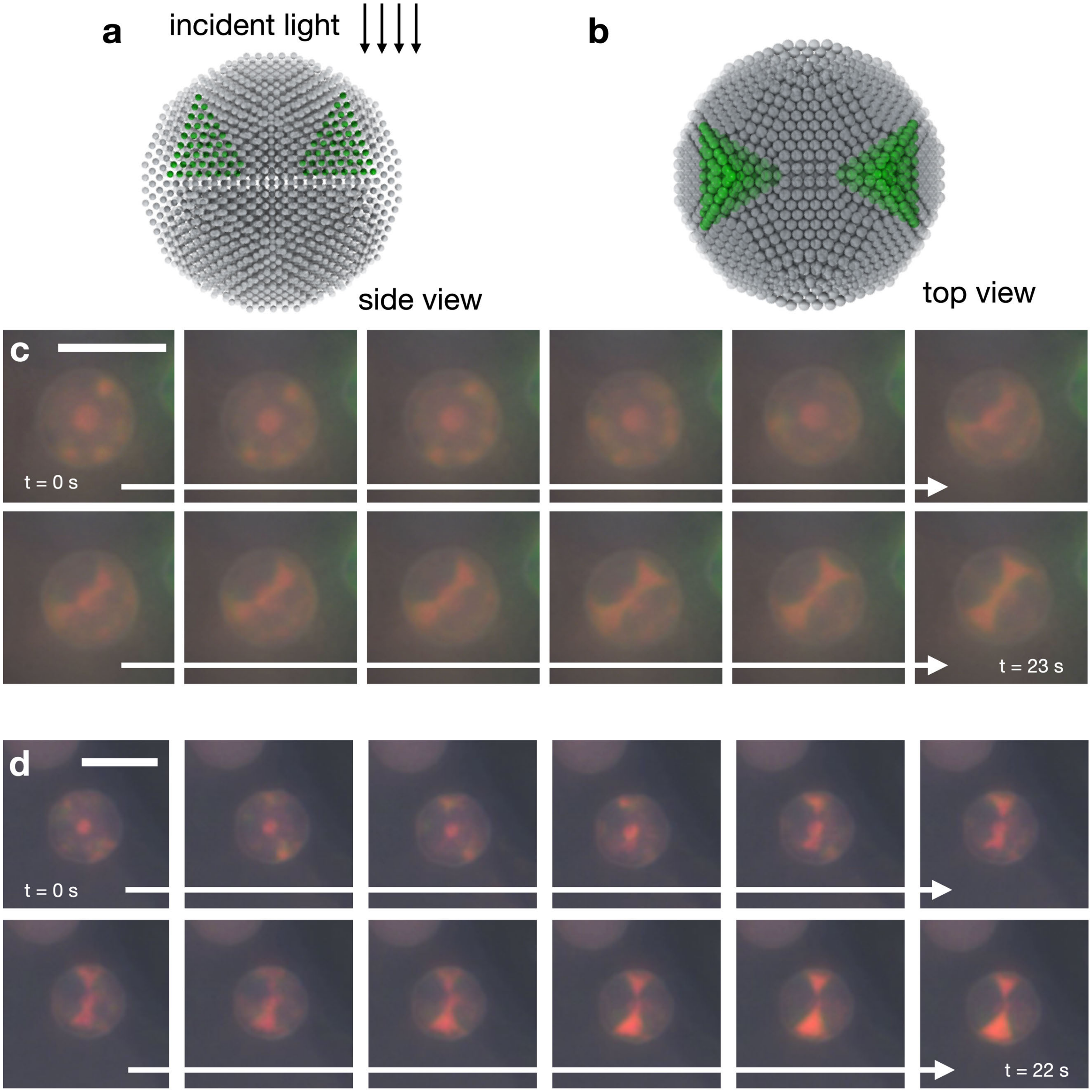

Supplementary Figure S18. Icosahedral colloidal cluster formation in drying droplets. **a,b**, Icosahedral cluster model aligning (111) planes of two tetrahedral grains (green) perpendicular to incident light giving rise to structural color with a bow-tie motif when viewed along a two-fold axis. **c**, Observation in real time under an optical microscope in reflection mode with an 125x objective (Supplementary Movie S9). The two triangles of the bow-tie motif form from the side. **d**, Another observation with a 40x objective (Supplementary Movie S10). The two triangles appear sequentially, the top triangle forms from the top side, the bottom triangle from the left side. Scalebar, 10 µm.

Supplementary References

1. Ino, S. Stability of Multiply-Twinned Particles. *J. Phys. Soc. Jpn.* **27**, 941–953 (1969).

2. Marks, L. D. Modified Wulff constructions for twinned particles. *J. Cryst. Growth* **61**, 556–566 (1983).

3. Wang, J., Mbah, C. F., Przybilla, T., Apeleo Zubiri, B., Spiecker, E., Engel, M. & Vogel, N. Magic number colloidal clusters as minimum free energy structures. *Nat. Commun.* **9**, 5259 (2018).

4. Wang, J., Sultan, U., Goerlitzer, E. S. A., Mbah, C. F., Engel, M. & Vogel, N. Structural Color of Colloidal Clusters as a Tool to Investigate Structure and Dynamics. *Adv. Funct. Mater.* **30**, 1907730 (2020).
