# Supplementary material for: Early-stage bifurcation of crystallization in a sphere
Source: Nat Commun. 2023 Aug 31;14:5299. doi: 10.1038/s41467-023-41001-6 (PMC10471623; doi:10.1038/s41467-023-41001-6)
Supplement: Supplementary file 3 — Description of Additional Supplementary Files [file 41467_2023_41001_MOESM3_ESM.pdf]

Description of Additional Supplementary Files

Supplementary Movie 1. Tilt series of X-ray images of a decahedral colloidal cluster consisting of 500 nm PS particles on solid substrate.

Supplementary Movie 2. Surface of 3D reconstruction of the decahedral colloidal cluster from X-ray tomography.

Supplementary Movie 3. Slices through the 3D reconstruction of decahedral colloidal cluster along the five-fold axis showing individual PS particles and defect plane.

Supplementary Movie 4. Simulation trajectory forming a decahedral cluster. Grains are shown with random color, fluid particles semi-transparent. Insets are the bond-orientational order diagram (bottom left) and the grain projection (bottom right). Three snapshots of this movie are analyzed in Figure 3a-c.

Supplementary Movie 5. Growth of decahedral colloidal cluster in emulsion droplet observed in real-time under optical microscope with 125x objective. Grains grow from one side to the other and form characteristic half-circle structural color pattern.

Supplementary Movie 6. Growth of decahedral colloidal cluster in emulsion droplet observed in real-time under optical microscope with 40x objective. Grains grow from one side to the other and form characteristic half-circle structural color pattern.

Supplementary Movie 7. Simulation trajectory forming an icosahedral cluster. Three snapshots of this movie are analyzed in Figure 3e-g.

Supplementary Movie 8. Simulation trajectory forming a defective cluster. The impossibility to form a complete icosahedron is not yet apparent, it affects only the final stage of droplet drying.

Supplementary Movie 9. Growth of icosahedral colloidal cluster in emulsion droplet observed in real-time under optical microscope with 125x objective. Individual grains grow sequentially to form characteristic bow-tie structural color pattern.

Supplementary Movie 10. Growth of icosahedral colloidal cluster in emulsion droplet observed in real-time under optical microscope with 40x objective. Individual grains grow sequentially to form characteristic bow-tie structural color pattern.
